# Vitamin D receptor gene polymorphisms are associated with breast cancer risk in a UK Caucasian population

**DOI:** 10.1054/bjoc.2001.1864

**Published:** 2001-07

**Authors:** D Bretherton-Watt, R Given-Wilson, J L Mansi, V Thomas, N Carter, K W Colston

**Affiliations:** Department of Oncology, Gastroenterology, Endocrinology and Metabolism, Department of Cellular Pathology, Medical Genetics Unit, St George’s Hospital Medical School; Duchess of Kent Breast Screening Unit, London, SW17 0RE

**Keywords:** vitamin D, receptor, human, polymorphism, breast cancer, caucasian

## Abstract

There is increasing evidence that vitamin D can protect against breast cancer. The actions of vitamin D are mediated via the vitamin D receptor (VDR). We have investigated whether polymorphisms in the VDR gene are associated with altered breast cancer risk in a UK Caucasian population. We recruited 241 women following a negative screening mammogram and 181 women with known breast cancer. The VDR polymorphism *Bsm*I, an intronic 3′ gene variant, was significantly associated with increased breast cancer risk: odds ratio *bb* vs *BB* genotype = 2.32 (95% CI, 1.23–4.39). The *Bsm*I polymorphism was in linkage disequilibrium with a candidate translational control site, the variable length poly (A) sequence in the 3′ untranslated region. Thus, the ‘L’ poly (A) variant was also associated with a similar breast cancer risk. A 5′ VDR gene variant, *Fok*I, was not associated with breast cancer risk. Further investigations into the mechanisms of interactions of the VDR with other environmental and/or genetic influences to alter breast cancer risk may lead to a new understanding of the role of vitamin D in the control of cellular and developmental pathways. © 2001 Cancer Research Campaign http://www.bjcancer.com


*British Journal of Cancer* (2001) **85**(2), 171–175
© 2001 Cancer Research Campaign
doi: 10.1054/ bjoc.2001.1864, available online at http://www.idealibrary.com on IDEAL®

### Vitamin D receptor gene polymorphisms are associated with breast cancer risk in a UK Caucasian population

**D Bretherton-Watt[1], R Given-Wilson[2], JL Mansi[1], V Thomas[3], N Carter[4] and KW Colston[1]**

[1]Department of Oncology, Gastroenterology, Endocrinology and Metabolism, [3]Department of Cellular Pathology and [4]Medical Genetics Unit, St George's Hospital Medical School; [2]Duchess of Kent Breast Screening Unit, London, SW17 0RE

**Summary** There is increasing evidence that vitamin D can protect against breast cancer. The actions of vitamin D are mediated via the vitamin D receptor (VDR). We have investigated whether polymorphisms in the VDR gene are associated with altered breast cancer risk in a UK Caucasian population. We recruited 241 women following a negative screening mammogram and 181 women with known breast cancer. The VDR polymorphism *Bsm*I, an intronic 3′ gene variant, was significantly associated with increased breast cancer risk: odds ratio *bb* vs *BB* genotype = 2.32 (95% CI, 1.23–4.39). The *Bsm*I polymorphism was in linkage disequilibrium with a candidate translational control site, the variable length poly (A) sequence in the 3′ untranslated region. Thus, the 'L' poly (A) variant was also associated with a similar breast cancer risk. A 5′ VDR gene variant, *Fok*I, was not associated with breast cancer risk. Further investigations into the mechanisms of interactions of the VDR with other environmental and/or genetic influences to alter breast cancer risk may lead to a new understanding of the role of vitamin D in the control of cellular and developmental pathways. © 2001 Cancer Research Campaign http://www.bjcancer.com

**Keywords**: vitamin D; receptor; human; polymorphism; breast cancer; caucasian

Breast cancer is the commonest cancer among women in the UK, with a lifetime risk of almost 1 in 10 (Anderson, 1992). While familial cancers account for around 5% of all breast cancers, the remainder appear to be the result of a multifactorial aetiology that includes a genetic component. Breast cancer is known to be strongly influenced by the hormonal milieu, and variation in genes that are responsive to such hormones are therefore possible candidates for increased risk. One potential target is the vitamin D receptor (VDR), a member of the steroid-hormone family of nuclear receptors, which are responsible for the transcriptional regulation of a number of hormone-responsive genes. The vitamin D receptor (VDR) is expressed in breast tissue, and patients with VDR-positive breast tumours have longer disease-free survival compared to those with receptor-negative tumours (Colston et al, 1989). The VDR ligand is the vitamin D metabolite, 1,25 dihydroxyvitamin $D_3$ (1,25-D), which has potent effects on cell growth and differentiation. Laboratory studies have demonstrated that 1,25-D and its analogues inhibit cell proliferation and promote apoptosis in breast cancer cells in culture (Chouvet et al, 1986; Eisman et al, 1989; James et al, 1995; Welsh, 1995). In animal models of breast cancer, vitamin D analogues delay tumour development and cause regression of established mammary tumours (Abe et al, 1991; James et al, 1998). Such evidence has led to the development of vitamin D analogues as potential new therapeutic agents (Bower et al, 1991; Gulliford et al, 1998) in breast cancer.

The gene encoding the VDR is known to contain a number of polymorphisms. A polymorphic start codon in the 5′ end of the gene (identified by the restriction enzyme *Fok*I) results in VDR proteins that differ in length by 3 amino acids. This polymorphism has been associated with increased breast cancer risk in African-American women (Ingles et al, 1997a). 3 sequences in the 3′ end of the gene (generating *Bsm*I, *Apa*I and *Taq*I restriction sites) are thought to be linked to a further polymorphism, the variable length poly (A) sequence in the 3′ untranslated region (3′ UTR). Such 3′UTR elements are candidate translational control sites, important in the post-transcriptional control of gene expression (Day and Tuite, 1998). An association between these 3′ polymorphisms and bone mineral density has been widely reported (Morrison et al, 1994; Cooper and Umbach, 1996). They have also been related to a number of other diseases including prostatic and breast carcinoma: in separate US studies, increased risk of prostate cancer has been associated with a long poly (A) allele (Ingles et al, 1997b), absence of *Taq*I (Taylor et al, 1996) and presence of *Bsm*I (Ma et al, 1998) restriction sites. An association has also been reported between breast cancer risk and the *Apa*I polymorphism (Curran et al, 1999), breast cancer progression and absence of the *Taq*I polymorphism (Lundin et al, 1999), and *Bsm*I genotype and increased risk of breast metastases (Ruggiero et al, 1998). These polymorphisms are thought to be in linkage disequilibrium in Caucasian populations, suggesting they are essentially looking at the same genotype (Ingles et al, 1997c).

This study was undertaken to assess whether VDR polymorphisms in both the 3′ and 5′ end of the gene are associated with breast cancer risk in a UK Caucasian population. In order to be as certain as possible that our control group is free of any breast cancer or precancerous changes that may go undetected in the general population, women were only recruited following a negative screening mammogram.

*Received 18 October 2000*
*Revised 26 March 2001*
*Accepted 27 March 2001*

*Correspondence to:* KW Colston

### METHODS

### Subjects

For both sample groups, written informed consent was obtained at the time of interview and sampling. The study was approved by the

St George's Hospital Medical School Ethics Committee. Recruitment criteria indicated that volunteers (i) were Caucasian and (ii) had had a recent mammogram. Women with family history of breast cancer were not specifically excluded from the study: the reported incidence was not different between our control and case groups and showed no relationship with VDR genotype.

### Control volunteers

Women ($n$ = 241) were recruited through the UK National Breast Screening Programme for South-West London. This provides for routine mammography of all women between the ages of 50–65 years at 3 yearly intervals, and 65+ years by self-referral. Currently, around 5% women screened are recalled to the Unit due to a technical problem with their mammogram, or for further investigation. Most of these women are subsequently found to be healthy and are discharged back into the Screening Programme. These women therefore have no detectable cancer at time of sampling, although 89 women had breast conditions not associated with breast cancer risk (26 had benign calcifications, 26 had fibrocystic disease and 37 had a fibroadenoma/other benign lump). The benign nature of these conditions was confirmed by cytology and/or radiological stability over time. Because of the nature of the Screening Programme, control women were in the age range 50–81 years, with a median age of 55.2 years, at the time of sampling. The age of the control group was therefore different from the case group. However, there were no age-related differences in VDR gene frequency between the oldest and youngest women. The majority of the control group (167 women, 69%) were postmenopausal and 126 women (52%) were current or past users of hormone replacement therapy (HRT).

### Breast cancer volunteers

Women ($n$ = 181) were recruited through the Combined Breast Clinic at St. George's Hospital. Women had a median age of 62.1 (range 29.0–91.0) years at the time of sampling. The median age at diagnosis was 57.2 (range 26.2–89.8) years, with a median time since diagnosis of 4.3 (range 0.4–27.5) years. Of this group, 149 women (82%) were postmenopausal, and 52 (29%) women were current/past users of HRT. All women had a surgical procedure (wide local excision or mastectomy) with or without post-operative radiotherapy.

The characteristics of the tumours were confirmed from histopathological records of core biopsy and/or resection specimens: 20 women had ductal carcinoma-in-situ (DCIS), 70 invasive ductal carcinoma (IDC), 77 both DCIS and IDC, and 14 invasive lobular carcinoma with or without lobular carcinoma in situ. Tumour grade data was available for 147 patients: 47 (32%) patients were classed as Bloom and Richardson Grade I, 58 (39%) patients as Grade II and 42 (29%) patients Grade III. Lymph nodes were taken from 140 patients. Of these, 108 (77%) had no lymph node involvement. Oestrogen receptor (ER) levels were determined for 167 patients, of whom 131 (78%) were ER positive. Adjuvant tamoxifen was given to 108 women, adjuvant chemotherapy to 20 women and a combination of both to 32 women. Since diagnosis, 21 women have had local recurrence, 3 women have had new primary breast tumours and 4 have developed metastatic disease.

### Analysis of VDR polymorphisms

A 10 ml blood sample was collected into a lithium heparin tube and used for extraction of DNA (QIAamp blood kit, Quiagen UK Ltd, W Sussex, England). Genomic DNA was amplified by PCR using specific primers as previously described (Morrison et al, 1994; Ingles et al, 1997b; Gross et al, 1998). For *Bsm*I and *Fok*I genotyping, PCR product was digested with the appropriate restriction endonuclease (New England Biolabs UK Ltd, Hertfordshire, England), separated by agarose gel electrophoresis and visualized by ethidium bromide staining. For both *Bsm*I and *Fok*I, genotypes were defined by capital letters in the absence of the restriction site (*B*, *F* respectively) and small letters where the restriction site was present (*b*, *f* ). For the poly (A) analysis, a 425 base pair PCR product was separated on a 6% PAGE-urea gel and visualized by silver staining (Silver Sequence[R], Promega UK, Southampton, England). Under these conditions, the poly (A) region resolves into 2 distinct populations, long (L, A18–A24) and short (S, A13–A17).

### Statistical analysis

The $\chi^2$ test was used to assess any association between VDR polymorphisms and breast cancer risk. Odds ratios and 95% confidence intervals were calculated to determine the risk of breast cancer associated with a given VDR genotype using the Clinistat Programme, devised by Professor Martin Bland, Dept. Public Health, St George's Hospital Medical School, London, UK. Allele frequencies were assessed for deviation from expected Hardy-Weinberg Equilibrium using the $\chi^2$ test.

### RESULTS

The frequency of VDR polymorphisms in our sample populations are shown in Table 1. We found a highly significant difference in genotype frequencies between patients and controls, such that the *bb* genotype was significantly over-represented in the patient population (Table 1). The odds of breast cancer for a woman of genotype *bb* were twice (OR = 2.32, 95% CI 1.23–4.39, Table 1) those for a woman of genotype *BB*.

The allele frequencies were similar to those reported in other Caucasian populations (Morrison et al, 1994; Houston et al, 1996; Harris et al, 1997; Ingles et al, 1997b; Kiel et al, 1997; Vandevyer et al, 1997; Ferrari et al, 1998). There was slight deviation from expected Hardy-Weinberg frequencies ($P$ = 0.05) for the *Bsm*I and poly (A) genotypes, but not the *Fok*I, in the control population only. Review and checking of our genotyping, including sequencing 12 random control DNA samples around the *Bsm*I site (data not shown), confirmed our genotyping. As the *Fok*I polymorphism was in Hardy-Weinberg equilibrium, we suggest that this result is an anomaly of our sample group.

The *Bsm*I polymorphism was found to be in strong linkage disequilibrium (410/419 samples genotyped, 98% agreement) with the variable length poly (A) sequence, such that *bb* genotype co-segregated with LL long poly (A). Independent analysis of breast cancer risk associated with the poly (A) genotype were similar to those associated with *Bsm*I (Table 1). No association was found between *Fok*I genotype and breast cancer risk (Table 1). The *Fok*I genotype was not in linkage disequilibrium with the poly (A) polymorphism (data not shown), suggesting that they segregate independently.

 © 2001 Cancer Research Campaign

**Table 1** VDR polymorphism frequencies

| | Controls *n* (%) | Allele frequency | Cases *n* (%) | Allele frequency | Odds ratio (95% confidence interval) |
|---|---|---|---|---|---|
| *Bsm*I | | | | | $\chi^2$ test, *P* = 0.0061** (d.f.=2) |
| *bb* | 69 (28.6) | *b* 0.56 | 78 (43.1) | *b* 0.66 | 2.32 (1.23–4.39) |
| *Bb* | 133 (55.2) | *B* 0.44 | 84 (46.4) | *B* 0.34 | 1.30 (0.70–2.39) |
| *BB* | 39 (16.2) | | 19 (10.5) | | 1.0 |
| Poly A | | | | | $\chi^2$ test, *P* = 0.0068** (d.f.=2) |
| LL | 67 (28.2) | L 0.56 | 76 (42.0) | L 0.66 | 2.46 (1.29–4.70) |
| LS | 132 (55.5) | S 0.44 | 87 (48.1) | S 0.34 | 1.43 (0.77–2.66) |
| SS | 39 (16.4) | | 18 (9.9) | | 1.0 |
| *Fok*I | | | | | $\chi^2$ test, *P* = 0.68 (d.f.=2) |
| *FF* | 86 (35.7) | *F* 0.60 | 72 (39.8) | *F* 0.62 | 1.17 (0.65–2.08) |
| *Ff* | 116 (48.1) | *f* 0.40 | 81 (44.8) | *f* 0.38 | 0.97 (0.55–1.71) |
| *ff* | 39 (16.2) | | 28 (15.5) | | 1.0 |

** statistically significant. d.f. = degrees of freedom.

We investigated whether any of the VDR polymorphisms were associated with particular clinical/pathological characteristics of our breast cancer group (Table 2). Data is shown only for *Bsm*I polymorphism, although all 3 genotypes were tested. Due to the small numbers of *BB* genotype, for $\chi^2$ test analysis data were pooled from *BB* and *Bb* genotypes. There was no association between any VDR genotype and either ER expression of the tumour or lymph node involvement. However, there was a significant association between the tumour grade and *Bsm*I genotype, with an excess of *bb* genotype in those tumours of grades II and III. Similar results were seen for poly (A) genotype (results not shown). This suggests that the *Bsm*I/poly (A) is associated with tumour progression in addition to disease risk.

### DISCUSSION

We have found a highly significant association between the risk of breast cancer and the 3′ VDR gene polymorphisms, *Bsm*I and variable length poly (A) microsatellite, in a UK Caucasian population. This study has added to the increasing evidence for a role of VDR gene polymorphisms in the disease process: polymorphisms have been widely associated with disorders of bone (Morrison et al, 1994; Cooper and Umbach, 1996), and there is increasing evidence for an association with prostate and breast cancer (Taylor et al, 1996; Ingles et al, 1997b; Ma et al, 1998; Ruggiero et al, 1998; Curran et al, 1999; Lundin et al, 1999).

In Caucasian populations, there is thought to be strong linkage disequilibrium between the 3′ polymorphisms, *Bsm*I, *Apa*I, *Taq*I and the variable length poly (A), such that only 2 haplotypes are commonly observed: *baTL* and *BAtS* (Morrison et al, 1994; Ingles et al, 1997c). It is the *baTL* haplotype that appears to be associated with increased risk of breast and prostate cancer (Taylor et al, 1996; Ingles et al, 1997b; Ma et al, 1998; Ruggiero et al, 1998; Curran et al, 1999; Lundin et al, 1999), while the *BAtS* haplotype is associated with increased risk of osteoporosis (Morrison et al, 1994; Cooper and Umbach, 1996). Not all studies have shown such associations: increased breast cancer risk among Latina women in the United States was associated with SS/*BB* genotypes (Ingles et al, 2000), and no association was found between the VDR *Taq*I polymorphism and breast cancer risk in Caucasian women in the UK (Dunning et al, 1999).

There are difficulties, however, in equating different polymorphisms between studies. For example, the strength of linkage disequilibrium can vary significantly between populations (Ingles et al, 1997c), resulting in misclassification of the ‘at-risk’ locus. VDR allele frequencies may also vary within Caucasian populations: of

**Table 2** Further analysis of cancer group in relation to *Bsm*I genotype

| Risk factor | | | | $\chi^2$ test for *Bsm*I genotype |
|---|---|---|---|---|
| ER status of tumour | *ER+ve* | *ER–ve* | | |
| *bb* | 59 | 14 | | $\chi^2$ test, *P* = 0.51 (d.f.=1) |
| *Bb/BB* | *72* | *22* | | |
| total | 131 | 36 | | |
| Lymph node involvement | *Node –ve* | *Node+ve* | | |
| *bb* | 45 | 15 | | $\chi^2$ test, *P* = 0.60 (d.f.=1) |
| *Bb/BB* | *63* | *17* | | |
| total | 108 | 32 | | |
| Tumour Grade | *Grade I* | *Grade II* | *Grade III* | |
| *bb* | 15 | 32 | 16 | $\chi^2$ test, *P* = 0.043* (d.f.=2) |
| *Bb/BB* | *32* | *26* | *26* | |
| total | 47 | 58 | 42 | |

* statistically significant. d.f. = degrees of freedom.

*© 2001 Cancer Research Campaign* 

10 polymorphic genes assessed in a Finnish study, the prevalence of the 3′ *Taq*I polymorphism was significantly different from other Caucasian populations (Woodson et al, 1999). That the Caucasian population is heterogeneous with respect to the VDR gene is one possible explanation for such discrepancies between population-based VDR association studies.

Another difficulty with VDR polymorphisms studies is that, to date, no clearly defined functional effect of the different genotypes has been demonstrated. The *BB* genotype has been associated with elevated serum calcitriol levels (Morrison et al, 1994; Ma et al, 1998), and the *BAt* haplotype shown to have increased activity in a reporter gene assay (Morrison et al, 1994). There has been no conclusive demonstration of any genotype association with VDR mRNA levels: no effect (Mocharla et al, 1997; Gross et al, 1998), decreased (Carling et al, 1998) and increased (Verbeek et al, 1997) expression of mRNA have all been reported.

One problem is the nature of the polymorphisms: the *Bsm*I and *Apa*I polymorphisms are intronic, while *Taq*I leads to a silent codon change. As such, they do not apparently lead to any change in either the transcribed mRNA or translated protein. It has been suggested that they are markers for the poly (A) sequence: such elements in the 3′ UTR of genes are thought to be important in post-transcriptional control of gene expression (Day and Tuite, 1998), either by altering mRNA stability or the interaction of the mRNA with the translational apparatus. There has been no evidence to date, however, that the poly (A) element has such a role – attempts to demonstrate an effect on mRNA stability were negative (Durrin et al, 1999). Further work is clearly necessary to try and identify functional mechanisms for the observed effects of the VDR polymorphisms.

Finally, while VDR polymorphisms may influence disease risk, such genetic factors cannot be dissociated from environmental influences. Breast cancer is strongly influenced by hormonal factors, in particular oestrogen. We found no association between VDR genotype and ER status in our group of largely post-menopausal women. In contrast, Lundin et al (1999) found a trend towards increased survival in ER-positive, pre-menopausal women possessing the *tt* genotype. However, the relationship between VDR polymorphisms and breast cancer risk may be very different in pre- and post-menopausal women. Dietary influences may also be important in modulating risk: *bb* genotype has been associated with increased prostate cancer in elderly men who are also deficient in vitamin D (Ma et al, 1998). It would be interesting to determine the extent of vitamin D deficiency in conjunction with VDR polymorphisms and breast cancer risk.

In conclusion, this study has provided additional support for a significant association between VDR gene polymorphisms and the risk of breast cancer. The potent actions of vitamin D and its analogues as anti-proliferative and pro-differentiation agents in breast cancers (Chouvet et al, 1986; Eisman et al, 1989; Abe et al, 1991; James et al, 1995; Welsh, 1995; James et al, 1998) have led to the suggestion that endogenous levels of active vitamin D are a factor in the development and progression of breast cancers. However, the ability of 1,25-D to act at the cellular level will be influenced by levels and activity of the VDR. While vitamin D and its analogues are being developed as preventative and/or treatment agents in breast cancer, the assessment of VDR polymorphisms may be vital in the identification of at-risk groups and strategies for targeting and intervention.

### ACKNOWLEDGEMENTS

This research was supported by the St George’s Hospital Special Trustees Research Fund. We would like to acknowledge Professor Martin Bland, Dept Public Health, St George’s Hospital Medical School, London and Dr EL Duncan, Wellcome Trust Centre for Human Genetics, Oxford for their statistical advice and discussions. We are grateful to Claire MacDonald for assisting with sample collection and Dr Petros Syrris for the DNA sequencing.

### REFERENCES

Abe J, Nakano T, Nishii Y, Matsumoto T, Ogata E and Ikeda K (1991) A novel vitamin D3 analog, 22-oxa-1,25-dihydroxyvitamin D3 inhibits the growth of breast cancer in vivo and in vitro without causing hypercalcaemia. *Endocrinology* **129**: 832–837

Anderson DE (1992) Familial versus sporadic breast cancer. *Cancer* **70**: (suppl): 1740–1746

Bower M, Colston KW, Stein R, Hedley A and Coombes RC (1991) Topical vitamin D analogue (calcipotriol) therapy in advanced breast cancer. *Lancet* **337**: 701–702

Carling T, Rastad J, Akerstrom G and Westin G (1998) Vitamin D receptor (VDR) and parathyroid hormone mRNA levels correspond to polymorphic VDR alleles in human parathyroid tumours. *J Clin Endo Metab* **83**: 2255–2259

Chouvet C, Vicard E, Devonee M and Saez S (1986) 1,25-Dihydroxyvitamin D3 inhibitory effect on the growth of two human breast cancer cell lines (MCF-7, BT-20). *J Steroid Biochem Mol Biol* **24**: 373–376

Colston K, Berger U and Coombes RC (1989) Possible role for vitamin D in controlling breast cancer cell proliferation. *Lancet* **I**: 188–191

Cooper GS and Umbach DM (1996) Are vitamin D receptor polymorphisms associated with bone mineral density? A meta analysis. *J Bone Min Res* **11**: 1841–1849

Curran JE, Vaughn T, Lea RA, Weinstein SR, Morrison NA and Griffiths LR (1999) Association of a vitamin D receptor polymorphism with sporadic breast cancer development. *Int J Cancer* **83**: 723–726

Day DA and Tuite MF (1998) Post-transcriptonal gene regulatory mechanisms in eukaryotes: an overview. *J Endocrinol* **157**: 361–371

Dunning AM, McBride S, Gregory J, Durocher F, Foster NA, Healey CS, Smith N, Pharoah PDP, Luben RN, Easton DF and Ponder BAJ (1999) No association between androgen or vitamin D receptor gene polymorphisms and risk of breast cancer. *Carcinogenesis* **20**: 2131–2135

Durrin LK, Haile RW, Ingles SA and Coetzee GA (1999) Vitamin D receptor 3′ untranslated region polymorphisms: lack of effect on mRNA stability. *Biochem et Biophysic a Acta* **1453**: 311–320

Eisman JA, Sutherland RI, McMenemy ML, Fragonas J-C, Musgrove EA and Pang GYN (1989) Effects of 1,25-dihydroxyvitamin D3 on cell cycle kinetics of T47D human breast cancer cells. *J Cell Physiol* **138**: 611–616

Ferrari S, Rizzoli R, Manen D, Slosman D and Bonjour J-P (1998) Vitamin D receptor gene start codon polymorphism (FokI) and bone mineral density: interaction with age, dietary calcium and 3′ end region polymorphisms. *J Bone Min Res* **13**: 925–930

Gross C, Musiol IM, Eccleshall TR, Malloy PJ and Feldman D (1998) Vitamin D receptor gene polymorphisms: analysis of ligand binding and hormone responsiveness in cultured skin fibroblasts. *Biochem Biophys Res Commum* **242**: 467–473

Gulliford T, English J, Colston KW, Menday P, Moller S and Coombes RC (1998) A phase I study of the vitamin D analogue EB 1089 in patients with advanced breast and colorectal cancer. *Br J Cancer* **78**: 6–13

Harris SS, Eccleshall TR, Gross C, Dawson-Hughes B and Feldman D (1997) The vitamin D receptor start codon polymorphism (FokI) and bone mineral density in premenopausal American black and white women. *J Bone Min Res* **12**: 1043–1048

Houston LA, Grant SFA, Reid DM and Ralston SH (1996) Vitamin D receptor polymorphism, bone mineral density, and osteoporotic vertebral fracture: studies in a UK population. *Bone* **18**: 249–252

Ingles SA, Haile RW, Henderson B and Coetzee GA (1997a) Polymorphisms in the 3′ and 5′ ends of the VDR gene are associated with breast cancer risk in African-American women. In: *Vitamin D: chemistry, biology and clinical applications of the steroid hormone* (AW Norman, R Bouillon, M Thomasset, eds) pp 813–815

Ingles SA, Ross RK, Yu MC Irvine RA, La Pera G, Haile RW and Coetzee GA (1997b) Association of prostate cancer risk with genetic polymorphisms in

 © 2001 Cancer Research Campaign vitamin D receptor and androgen receptor. *J Natl Cancer Inst* **89**: 166–170

Ingles SA, Haile RW, Henderson BE, Kolonel LN, Nakaichi G, Shi C-Y, Yu MC, Ross RK and Coetzee GA (1997c) Strength of linkage disequilibrium between two vitamin D receptor markers in five ethnic groups: implications for association studies. *Cancer Epidem Biomark Prev* **6**: 93–98

Ingles SA, Garcia DG, Wang W, Nieters A, Henderson BE, Kolonel LN, Haile RW and Coetzee GA (2000) Vitamin D receptor genotype and breast cancer in Latinas (United States). *Cancer Causes Controls* **11**: 25–30

James SY, Mackay AG and Colston KW (1995) Vitamin D derivatives in combination with 9-cis retinoic acid promote active cell death in breast cancer cells. *J Mol Endocrinol* **14**: 391–3947

James SY, Mercer E, Brady M, Binderup L and Colston KW (1998) EB 1089, a synthetic analogue of vitamin D3, induces apoptosis in breast cancer cells in vivo and in vitro. *Brit J Pharmacol* **125**: 953–962

Kiel DP, Myers RH, Cupples LA, Kong XF, Zhu XH, Ordovas J, Schaefer EJ, Felson DT, Rush D, Wilson PWF, Eisman JA and Holick MF (1997) The BsmI vitamin D receptor restriction fragment polymorphism (bb) influences the effect of calcium intake on bone mineral density. *J Bone Min Res* **12**: 1049–1057

Lundin A-C, Soderkvist P, Eriksson B Bergmann-Jungestrom M and Wingren S (1999) Association of breast cancer gene progression with a vitamin D receptor gene polymorphism. *Cancer Res* **59**: 2332–2334

Ma J, Stampfer MJ, Gann PH, Hough HL, Giovannuci E, Kelsey KT, Hennekens CH and Hunter DJ (1998) Vitamin D receptor polymorphisms, circulating vitamin D metabolites and risk of prostate cancer in United States physicians. *Cancer Epidem Biomarkers Prev* **7**: 385–390

Mocharla H, Butch AW, Pappas AA, Flick JT, Weinstein RS, De Tongi P, Jilka RL, Roberston PK, Parfitt AM and Manolagas SC (1997) Quantification of vitamin D receptor mRNA by competative polymerase chain reaction in PBMC: lack of correspondence with common allelic variants. *J Bone Min Res* **12**: 726–732

Morrison NA, Qi CJ, Tokita A, Kelly, Krofts, Nguyen TV, Sambrook PN and Eisman JA (1994) Prediction of bone mineral density from vitamin D receptor alleles. *Nature* **367**: 284–287

Ruggiero M, Pacini S, Aterini S, Fallai C, Ruggiero C and Pacini P (1998) Vitamin D receptor gene polymorphism is associated with metastatic breast cancer. *Oncol Res* **10**: 43–46

Taylor JA, Hirvonen A, Watson M, Pittman G, Mohler JL and Bell DA (1996) Association of prostate cancer with vitamin D receptor gene polymorphism. *Cancer Res* **56**: 4108–4110

Vandevyer C, Wylin T, Cassiman J-J, Raus J and Geusens P (1997) Influence of the vitamin D receptor gene alleles on bone mineral density in postmenopausal and osteoporotic women. *J Bone Min Res* **12**: 241–247

Verbeek W, Gombart AF, Shiohara M, Campbell M and Koeffler HP (1997) Vitamin D receptor: no evidence for allele-specific mRNA stability in cells which are heterozygous for the *Taq*I restriction enzyme polymorphism. *Biochem Biophys Res Comm* **238**: 77–80

Welsh JE (1995) Induction of apoptosis in breast cancer cells in response to vitamin D and antiestrogens. *Biochem Cell Biol* **72**: 537–545

Woodson K, Ratnasinghe D, Bhat NK, Stewart C, Tangrea JA, Hartman TJ, Stolzenberg-Solomon R, Virtamo J, Taylor PR and Albanes D (1999) Prevalence of disease-related DNA polymorphisms among participants in a large cancer prevention trial. *Eur J Canc Prev* **8**: 441–447

*© 2001 Cancer Research Campaign* 
